# Road manslaughter—or just the cost of progress?

**DOI:** 10.3134/ehtj.10.004

**Published:** 2009-10-27

**Authors:** L Mooren, R Grzebieta

**Affiliations:** Injury Risk Management Research Centre, The University of New South Wales, Sydney, Australia

## Abstract

Much to the frustration of road safety researchers, practitioners, and advocates, road deaths and injuries have not been widely accepted as a major public health threat. Currently, road trauma is one of the biggest killers and causes of serious and disabling injuries in the world. Although there has been considerable research on the causes of road injury and ways of mitigating the problem, there is still reluctance to systematically and sufficiently do what can be done to reduce this problem globally. This paper takes a historical review of the road trauma problem and responses to it. In examining developments in road transport and road injury, it is clear that the main impediment to reducing road deaths and injury has been a misguided preference of economic advancement over public health risk management. It is misguided because road trauma has impeded and does still impede the capacity of economies to develop. The challenge for societies now is to look at this false dichotomy—that of road development and motorisation versus road safety—and begin to make the right choices in favour of human society advancement through the development and management of safe road-traffic systems. A new ‘Safe Systems’ approach is emerging in Australia and spreading globally as a guiding principle for road safety. The evolution of this approach is traced and illustrated in this article. The need for finding ways to engender a stronger global political commitment to road safety is demonstrated.

## Introduction

The first recorded automobile fatality occurred in Ireland, in 1869.^1^ The event was described as a ‘public scourge and a private tragedy’. The coroner was moved to say, ‘This must never happen again’. But then in 1899, Henry Bliss was killed when struck by a taxi in the United States while alighting from a streetcar. Ward and Warren^2^ point out that road deaths came to be seen as a social class issue in the early days of ‘horseless travel’, as it was usually the poor and working classes that were killed by motor vehicles driven by wealthier people.

Nearly a century later, after World War II in 1947, JS Dean wrote a book entitled, ‘Murder Most Foul: a study of the road deaths problem’. He concluded that ‘The ‘reconstruction of Britain will indeed be a dismal failure if it includes as a permanent feature of the national life the killing and maiming of a quarter of a million, or more, of persons every year on the roads…there is no reason for failure…all that is needed is the will to act.’^3^

Road safety is a political issue—and has been for a long time. Dean believed that increases in road deaths were directly related to the rise of fascism, pointing to the fact that Nazi Germany and Mussolini's Italy had the highest per-vehicle rate of road fatalities in 1934. He explains that, in these countries, the motor interests were the biggest supporters of Hitler and Mussolini. Dean illustrates how motor interests were protected in all road safety efforts by targeting the behaviour of vulnerable road users through education and punitive actions alone.

The typical Nazi government responses to the problem were to introduce fines, collectable on the spot for ‘careless walking’, and also for ‘endangering traffic (while walking in the road)’, and for riding a bicycle two abreast. Dean lamented the observation that Britain was also influenced strongly by motor interests, citing many examples of media comments in the mid-1930s about the imposition of a speed limit: that restrictions on speed would ‘fatally damage the motor industry’.

The politics of road safety have manifested in many forms. In Australia, bicycle groups organize ‘critical mass’ demonstrations, disrupting traffic during evening peak hours in metropolitan areas, to lobby for better and safer road space. Their perception is that road authorities are entirely focused on the needs of motor vehicles (http://www.criticalmass. org.au/).

At a global level, in fast-growing economies such as Vietnam and China, road safety is sidelined in favour of rapid road infrastructure development. In practice, the historical trend of increasing road deaths accompanying road development and motorisation has not been simply due to greed or deliberate acts by one stakeholder group at the expense of others. Rather, it has been a corollary to general socioeconomic trends with a pervading impetus towards modernisation and mobility.

## The problem

Around 3000 people each day or 1.2 million people each year are dying on the world's roads, and 50 million are injured.^4^, ^5^ Road-traffic injury is now the number one killer of young people aged between 10 and 24 years, of whom 96% are dying in developing countries.^6^ The World Health Organization (WHO) has also estimated that road fatalities and serious injuries will rise by 65% by 2020, that deaths resulting from road crashes will exceed those from HIV, malaria, and tuberculosis, and that road accidents are predicted to become the third leading contributor to the global burden of disease and injury. In fact, in a report published in 2003, the WHO^7^ categorised road-traffic crashes as the ‘hidden epidemic’ and as a much overlooked growing threat.

One becomes acutely aware of the magnitude and threat to communities when looking at the total number of deaths that occur in any country as a result of a traffic crash, and comparing it with the number of deaths resulting from all the wars and disasters its citizens have suffered. For example, the total number of fatalities Australia has suffered in all wars to date is around 103,000, of which only 36,000 occurred since 1925 (source: Australian War Memorial (http://www. awm.gov.au/research/infosheets/war_casualties.asp)). Added to this number should be the number of Australians who have died as a result of natural and human-caused disasters (fires, bridge collapses, bombings, etc.)—only around 1000. This total can then be compared with the ∼171,000 fatalities in total resulting from all road crashes since records began in 1925. This is almost double the number accumulated over a shorter period.

The figures contrast in a similar way for the United States. Around 1.8 million road fatalities (National Highway Traffic Safety Administration (NHTSA), US Department of Transportation, Traffic Safety Facts 2004; http://www-nrd. nhtsa.dot.gov/Pubs/TSF2004.PDF) have been recorded to date and since only 1966, compared with the ∼1.4 million fatalities from all wars, including the US civil war and disasters that include heat waves, hurricanes, floods, and bombings (Death Tolls for the Man-made Megadeaths of the 20th Century; http://users.erols.com/mwhite28/warsusa. htm#USWar). In 2000, fewer than 4000 people were killed in the Twin Towers terrorist attack in New York City, but more than 40,000 Americans are killed in road crashes every year. Yet, the US Government's attention towards anti- terrorist initiatives far outweighs the attention given to road safety. Indeed, when the casualties of wars and disasters are compared with those from traffic crashes for any developed nation, it becomes obvious that traffic crashes are a much greater risk to the public's health and well-being.

Moreover, the incidence and severity of road crashes is somewhat more predictable and preventable than are other causes of injury. Much more so than natural disasters, where magnitude and location are difficult to predict, and wars, where injury is intentional, road trauma is known to be caused by certain characteristics of roads, vehicles, and behaviours—all of which can be ameliorated.

Historical lessons also point to social and economic trends that are associated with sharp increases in road trauma. As motorised road travel exposure increases, so does road fatality risk—if nothing is done to prevent injuries from increased risk of motor vehicle use. Indeed, Australian road fatalities rose steeply during years of rapid post-war motorisation between the 1940s and the 1960s.

## Australian motorisation and road fatalities

**Figure 1:**
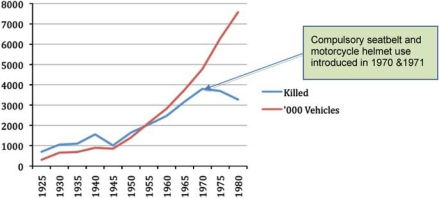
Road crash casualties and rates, Australia, 1925–1980, adapted from data source.^8^

However, the introduction of seatbelt and helmet laws in 1970 and 1971 began to curb this upward trend (Figure 1), showing that motorisation does not have to be accompanied by increasing death rates.

**Figure 2:**
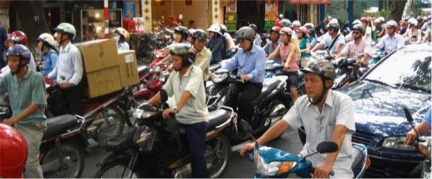
Traffic in Hanoi (photo: courtesy of the Asia Injury Prevention Foundation).

Sadly, it has taken Vietnam 36 years to learn and apply this lesson. A mandatory helmet law was introduced in December 2007, and achieved 95% compliance virtually overnight (Figure 2), accounting for the estimated saving of 1000 lives in 2008 (presented by Greig Craft, President, Asia Injury Prevention Foundation, at the NIOSH International Conference on Road Safety at Work, Washington, DC, USA, February 2009).

Road deaths are not an inevitable cost of economic development or motorisation. Simple measures such as introducing and enforcing compulsory helmet and seat belt laws can make a large difference in the trauma that comes with motorisation.

**Figure 3:**
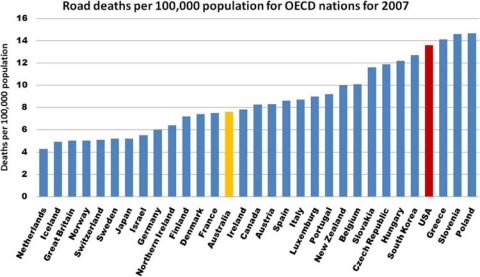
International benchmarking road fatality rates per 100,000 population for 2007 (data source: Department of Infrastructure, Transport, Regional Development and Local Government; http://www.infrastructure.gov.au/roads/safety/publications/2009/rsr_05.aspx—accessed November 2009) and WHO^9^ (http://www.who.int/violence_injury_prevention/road_safety_status/2009).

Moreover, reducing road injuries is not as simple as assuming that richer countries are more able to achieve better road safety outcomes. There is much variance in road safety achievements within the community of more economically advanced countries. The Netherlands, with a death rate at 4.3 per 100,000 people, compares favourably with Australia at 7.6. However, the rate in the United States (of 13.6) is three times the Dutch rate and is close to double the Australian rate. It is interesting to note that the US traffic fatality rate per 100,000 population is ranked 28th out of 30 OECD countries, with only Greece (14.1), Slovenia (14.6) and Poland (14.7) having slightly higher rates (see Figure 3).

The road fatality rates per 100,000 population indicate an individual's chances of dying from a road crash without explaining their exposure to risk. Although the nature of road injury risk might be more obvious in China and India, where the fatality rate is 5.62 and 14.5 per 10,000 vehicles, respectively, compared with the Netherlands or Australia, at 0.48 and 0.78 per 10,000 vehicles, respectively,^9^ the quantum of exposure is possibly less for China and India, at 9.2 and 16.1 persons per vehicle, respectively, than it is in countries that have achieved a high level of motorisation, at 1.4 and 1.8 persons per vehicle for Australia and the Netherlands, respectively, and where people are routinely exposed to high-speed traffic.

**Figure 4:**
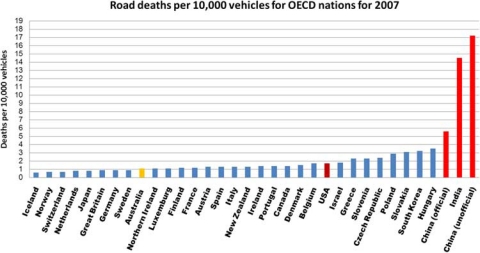
International benchmarking road fatality rates per 10,000 vehicles for 2007. (The majority of the values shown were determined from 2007 data. Where data were missing for 2007, the value from the closest year was used; that is, for Canada only 2006 data were available and hence were used.)^6^

China and India, with their massive development and thirst for automotive mobility, have suffered enormous road casualties. The official road fatality numbers for China currently stand at around 81,649 annually or 223 deaths per day, and for India it is 105,725 per annum. The official figures released by China have been disputed as under- reporting the actual number of fatalities, which is suggested to be nearly 250,000 (http://www.car-accidents.com/country-car- accidents/china-car-accidents-crash.html and http://www.wpro. who.int/china/sites/injury_prevention/ (accessed November 2009)). Figure 4 shows that when data are presented in terms of 10,000 registered vehicles, both India and China display particularly poor road safety records.

However, although the United States has a substantially reduced rate of traffic deaths per 10,000 vehicles compared with India and China, it still ranks poorly against comparable nations. This reflects a difference in approach from that of better-performing, though less wealthy, nations. Northern European nations have the lowest rates of road fatalities. (Although Japan is shown in Figure 3 as having a comparable rate to the countries with the lowest fatality rates, it uses a different definition of road fatalities than the ‘death within 30 days of the crash’ OECD standard.). The reasons for this may lie in cultural and historical differences.

India and China still have relatively low rates of motorisation per capita due to the unaffordability of motor vehicle ownership by much of their population. Ironically, relative impoverishment is perhaps containing road trauma levels so long as household incomes restrain the ability of most families to privately own motor vehicles. But at the same time, the road trauma problem is a massive burden on healthcare services; it also hampers the ability of these countries to advance economically. This is because road trauma—more so than other threats to public health—affects young productive males disproportionately.^10^

**Figure 5:**
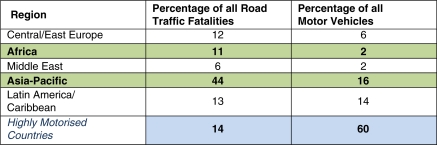
Road fatalities and vehicles: regional distribution (source: adapted from Jacobs *et al*.^11^).

Generally, as shown in Figure 5, road fatality rates in some world regions have disproportionate shares of road fatalities compared with their levels of motorisation.

Current trends suggest that highly motorised countries are reducing their road deaths, while in African and Asian countries they are increasing. Indeed, motorisation and road travel rates have continued to increase in high-income countries as well, equally or more so than in developing countries, while their fatality rates are dropping. It is a different story with developing countries. Road deaths are expected to increase by 80% in the Asia-Pacific and in parts of Sub-Saharan Africa between 2000–2020.

## What causes road deaths and injury?

At the most basic level of analysis, road injury is caused by impact on the human body of forces that the body cannot sustain without damage. Further, injury epidemiology identifies a range of factors that contribute to the crash event and the resulting impact on the human body.

Initially, the response to road deaths was to try to educate the masses about how to properly use the road. The assumption was that the problem was a knowledge and skill deficit among drivers—to drive safely—and among pedestrians and pedal cyclists—to keep out of the way of motor vehicles. Gradually, training, testing, and licensing processes emerged in countries where motor vehicle use was growing.

Later, some attention was directed to the motor vehicles themselves, but this was not very effective until some American legal specialists led by Ralph Nader took on the car industry with lawsuits focusing on the intrinsic, unacceptable, and unsafe features of cars. In 1965 and 1966, public pressure grew in the United States to increase the safety of cars, culminating with the publication of Ralph Nader's book, *Unsafe At Any Speed* 12 and the National Academy of Sciences’ *Accidental Death and Disability: The Neglected Disease of Modern Society*.^13^

In 1966, the US Congress held a series of highly publicized hearings regarding highway safety, and passed legislation to make installation of seat belts mandatory; it also created several agencies that would eventually be known as the NHTSA.^2^

**Figure 6:**
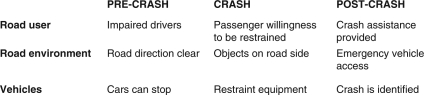
Haddon Matrix with sample injury factors (adapted from Haddon^14^).

At the same time, the ‘scientific method’ of analysing road injury causation was embraced in the 1960s following the work of Dr William Haddon, an injury epidemiologist. Haddon's injury analysis method called for identification of contributing factors before, during, and after the crash event, grouping them into three categories: vehicle, road environment, and human (as illustrated in Figure 6).

This was the springboard for a more systematic analysis of road injury. Injury specialists, particularly in the Western world, began to adopt this method. Biomechanics looked at vehicle features that contributed to more and more severe injuries, civil engineers looked more closely at road environment features, and behavioural scientists looked at unsafe road behaviours contributing to crashes and injuries. A more comprehensive and strategic approach emerged, boosting the ability of injury practitioners to direct their attention to the most important issues for road safety.

**Figure 7:**
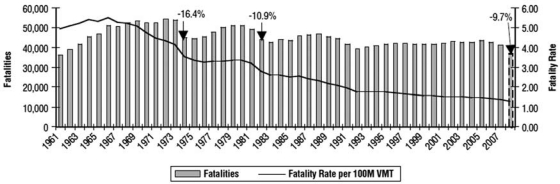
US fatalities and fatality rates per 100 million VMT from 1961 to 2008 (reproduced from NHTSA, 2008 Traffic Safety Annual Assessment—Highlights. Available at www-nrd.nhtsa.dot.gov/pubs/811172.pdf).

This has resulted in more favourable trends in road injury (http://www.fhwa.dot.gov/policyinformation/statistics/2007), especially considering the exposure to road injury risk (Figure 7), measured by road fatality rate per 100 million vehicle miles.

## Australian road safety successes

**Figure 8:**
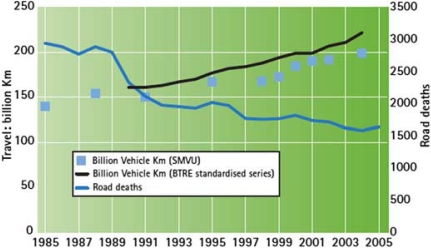
Comparison of road death numbers with road travel growth (reproduced from ATC^15^).

Australian road travel exposure has continued to increase since the post-war period, but through concerted efforts, road fatalities have been reduced substantially as shown in Figure 8.

The scientific method, together with a political commitment to invest in road safety, provided a solid and more effective base of knowledge upon which to build comprehensive road safety programs in Australia. Moreover, a key feature of Australian road safety that emerged was that institutional arrangements and collaborations between government agencies are vital to road safety effectiveness.

**Figure 9:**
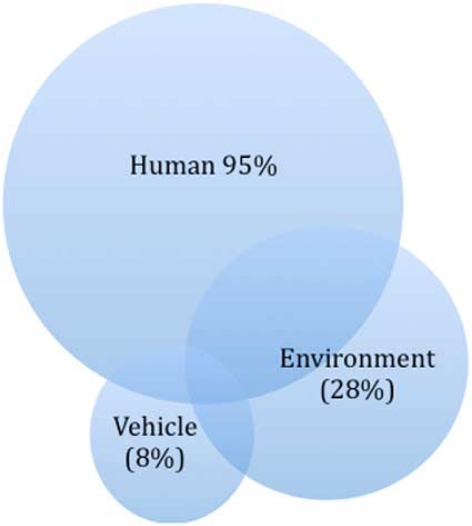
Road injury factors—Source: adapted from Morgan *et al*.^16^

Studies using the Haddon Matrix began to show that human factors were more prevalent among causes of motor vehicle crashes than other types of factors (see Figure 9). Indeed, some studies found that in 95% of fatal road crashes human factors were involved.

Although studies show that in 28% of fatal crashes road environment factors are involved, and in 8% vehicle factors are involved, in practice there are usually a number of factors that contribute to road crash outcomes. As knowledge has accumulated about specific risk factors, and on how to address them, effective road, vehicle and behavioural ‘countermeasures’ have been developed and implemented—with a great deal more success than had been achieved before this systematic and strategic approach had been developed.

In Australia, national and state government road safety authorities develop and implement programs to address the road safety issues. Road authorities, police, and sometimes health and education sectors or other interested bodies have been involved in strategies with increasingly coordinated actions. For example, road authorities and other bodies have been conducting public education campaigns that complemented police traffic enforcement operations. The most notable of these types of campaigns is perhaps the random breath testing anti-drink drive programs conducted in Australia from the early 1980s. These campaigns achieved some of the most dramatic reductions in road fatalities ever seen. Moreover, the holistic *strategic* approach to road safety is believed to be effective as illustrated by the general downward trend in road trauma.

At the same time, road environment safety improvements as well as vehicle and equipment safety improvements are being pursued. A ‘blackspots program’, targeting road locations with high crash involvements, and road safety audit programs are systematically addressing road environment risks. Vehicle and equipment (e.g. helmets) safety improvements are being more rigorously pursued too. Funding is being invested in road safety on the basis of ‘balancing safety and mobility’.

But in the mid-1990s, Sweden and the Netherlands began to question the notion of ‘balancing’ safety and mobility objectives. In these jurisdictions, the governments took the view that if human lives and limbs can be saved, they should be, regardless of other private and public interests.

Thus, a new *political* position in road safety was born—the *ethical approach*. The Swedish *Vision Zero* and Dutch *Sustainable Safety* policies tipped the balance toward safety over mobility.

Australia has adopted a ‘Safe Systems’ approach in an attempt to further reduce road fatalities. It is based on European models, in which the central road design and management criteria are focused on human injury tolerance to impact force; the models were adapted from Sweden's ‘Vision Zero’ strategy.^17^ This approach is preferred over the more traditional US cost–benefit-based templates, which are designed to focus on traffic efficiency, are car-centric, and are based on open and expansive road systems that readily lend themselves to abuse by facilitating excessive speeding and poor crash outcomes.

Safe Systems is based on the acknowledgement that humans make errors, but that the road-traffic system should be designed to compensate for that error so that road users will survive the consequences of their mistakes.^18^ Inherent in the Safe Systems approach is the commitment by the system owner or manager to do all that is possible to provide and manage a product that will not harm its users.

In a Safe System, if a road user travels in accordance with all traffic laws, on a safe road in a safe vehicle, but finds through no fault of their own that they become involved in a crash, the crash must be survivable and not result in long-term health loss. Similarly, if a driver makes an error, for example, falls asleep at the wheel and speeds, the system should react either actively or passively to alert and change the driver's behavior to minimize the consequences of the error. In other words, a driving error is corrected through systemic controls or, in the event of a crash, the forces harmful to human health are minimised.

Similarly, the regulatory system should function with appropriate responsive enforcement feedback. Any high-risk error such as speeding and drink driving should be strongly discouraged, be portrayed as socially unacceptable, and the system should allow for rehabilitation. Thus, all road user training and behaviour management, vehicle development and regulation, road design and traffic management systems should be governed and filtered according to this paradigm.

However, despite the acknowledgement that ‘the effective strategies for preventing or reducing crashes and injuries are well known’,^4^ a global paradigm shift requires efforts to shift political priorities. Indeed, the World Report on Road Traffic Injury Prevention^5^ specifically identifies actions to build up political will as a key requirement in global and local road safety efforts.

At a meeting in March 2008, the United Nations General Assembly passed a resolution (see http://www.un.org/News/ Press/docs/2008/ga10694.doc.htm) calling for the first global Ministerial Conference on road safety, in an effort to reduce the rapidly growing death toll on the world's roads. The conference will be hosted by the Russian Federation in 2009, and will be facilitated by the Commission for Global Road Safety with participation from ministers in the transport, health and financing areas of governments. The initiators are calling for a decade of road safety action (see http://www. makeroadssafe.org/news/2008/first_un_ministerial_summit_ on_global_road_safety_approved.html).

However, as King,^19^ Mohan^20^ and others have indicated, improving road safety in developing countries is not a simple matter of transplanting Western practices. They argue that to be successful, transfer of a road safety intervention must take into account the institutional, economic and social/cultural environment of the target jurisdiction. Moreover, an intervention is more likely to be successful if there is an active local response within a country, with stakeholders working in partnership to develop and carry out the intervention.

## Discussion and conclusions

At this point in history, human societies have the scientific knowledge and the technology to effectively eliminate road injury. The challenge is to fully embrace the opportunity. What is needed is a concerted effort to develop a global culture of road safety—one that embraces the Safe System principle irrespective of the stage of economic and road infrastructure development in a particular country.

The experience, especially in Australia, northern Europe and the United States, demonstrates that effective solutions to road injury risk can be implemented. The problem is that we do not fully understand the reasons for the apparent complacency of governments that fail to embrace the road safety problem.

In 1999, in initiating the formation of the Global Road Safety Partnership, James Wolfensohn, President of the World Bank, said that ‘road safety is an issue of immense human proportions. It is also an issue of equity. Road safety very much affects poor people.’ Yet, in 2000, no mention of road safety was included in the much-celebrated United Nations Millennium Development Goals (see http://www. un.org/millenniumgoals/).

Hence, although the efforts to share scientific knowledge and road safety management capacity is important for improving global road safety outcomes, more social science research is needed to develop effective strategies for developing community and political commitment to road safety action.
